# Impaired Cardiorespiratory Fitness in Type 1 Diabetes Is Associated With Metabolic Inflexibility and Specific Factors According to Sex

**DOI:** 10.1111/1753-0407.70164

**Published:** 2025-12-14

**Authors:** Liliana Muñoz‐Hernandez, Jesus Paez‐Mayorga, Jesus Flores‐Brito, Leticia Lopez‐Carreola, Sebastian Zamora‐Gutierrez, Daniel Laguna‐Arellano, Guadalupe Mixcoatl‐Galicia, Perla Alpizar‐Chacon, Eunice Barbosa‐Meillon, Mauro Morales‐Velasco, Erick Reséndiz‐Carrillo, Ivette Cruz‐Bautista, Roopa Mehta, Carlos A. Aguilar‐Salinas, Paloma Almeda‐Valdes

**Affiliations:** ^1^ Metabolic Diseases Research Unit, National Institute of Medical Sciences and Nutrition Salvador Zubirán Tlalpan Mexico; ^2^ The Secretariat of Science, Humanities, Technologies an Innovation (SECIHTI) Mexico City Mexico; ^3^ Department of Endocrinology and Metabolism National Institute of Medical Sciences and Nutrition Salvador Zubirán Mexico City Mexico; ^4^ School of Medicine and Health Sciences, Tecnologico de Monterrey Mexico City Mexico; ^5^ Scholarship Holder of the General Directorate of Quality and Health Education, Ministry of Health México City Mexico

**Keywords:** body composition, cardiopulmonary exercise test (CPET), cardiorespiratory fitness, indirect calorimetry, resting energy expenditure, type 1 diabetes

## Abstract

**Background:**

People with long‐term type 1 diabetes have reduced cardiorespiratory fitness (CRF). We aimed to assess the influence of body composition, energy substrate use, and blood glucose control on cardiopulmonary exercise test performance in subjects with uncomplicated, long‐term type 1 diabetes.

**Methods:**

Observational, cross‐sectional study. Subjects with and without type 1 diabetes, paired by sex and age, underwent treadmill cardiopulmonary exercise test, bioelectrical impedance analysis, indirect calorimetry at rest and during exercise. We used the *t*‐pair test; multivariate linear regression models and mediation analysis were used to evaluate determinants of CRF.

**Results:**

54 cases and 54 controls (52% female) were evaluated. The age was 39 (28–46) years, duration of disease 21 ± 10 years. HbA1c 7.9 (7.3–8.7). The baseline physical activity, resting energy expenditure, respiratory quotient and body composition were similar between groups. VO2max was 32 ± 9.2 versus 39 ± 7.9 mL/kg/min (*p* < 0.01) for cases and controls. Maximum carbohydrate oxidation was 809 (614–1174) versus 1082 (863–1454) (*p* < 0.01), respectively. In women 73% and 25% of the effect of diabetes status on VO2max were mediated by carbohydrate oxidation and heart rate reserve, respectively. In men 78% and 57% of the effect of diabetes status on VO2max were mediated by carbohydrate oxidation and phase angle, respectively.

**Conclusions:**

Type 1 diabetes group had an altered CRF with lower carbohydrate oxidation. This suggests altered metabolic flexibility due to low substrate availability that could explain the earlier fatigue during intense exercise. There were different determinants of VO2max in persons with diabetes according to sex.

## Graphical abstract

Design and main findings of the study.

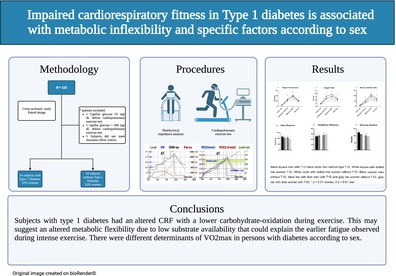

**Summary**

- The mechanisms that prompt VO2max reduction in persons living with T1D are mediated by carbohydrate oxidation for both sexes.
- Phase angle in men while heart rate reserve in women were specific determinants that mediated VO2max in the T1D population.

**Abbreviations**
6MWT: 6‐min walk test
ALT: alanine aminotransferase
ASP: autonomic symptom profile
AST: aspartate aminotransferase
AT: anaerobic threshold
ATP: adenosine triphosphate
BIA: bioelectrical impedance analysis
BMI: body mass index
bpm: beats per minute
BR: breathing reserve
CAN: cardiovascular autonomic neuropathy
CHO: carbohydrates
CHO‐ox: carbohydrate‐oxidation
Cm: centimeter
CO2: carbon dioxide
COMPASSS 31: composite autonomic symptom score—31
COPD: chronic obstructive pulmonary disease
CPET: cardiopulmonary exercise test
CRF: cardiorespiratory fitness
CV: cardiovascular
CVD: cardiovascular disease
DBP: diastolic blood pressure
ECG: electrocardiogram
ECW: extracellular water
FFM: fat‐free mass
FFMI: fat‐free mass index
FM: fat mass
G0: fasting glucose
HbA1c: glycosylated hemoglobin
HDL‐c: high‐density lipoprotein cholesterol
HR: heart rate
IPAQ‐9: International Physical Activity Questionnaire 9
Kcal: kilocalories
Kg: kilogram
LDL‐C: low‐density lipoprotein cholesterol
Max: maximum effort
MET: metabolic equivalent of task
METS‐IR: metabolic score insulin resistance index
METS‐VF: metabolic score for visceral fat
MH: metabolic health
N2: nitrogen
O2: oxygen
PCA: principal component analysis
Pred: predicted
PuO2: oxygen pulse
PuO2max: maximum oxygen pulse
QUICKI: quantitative insulin‐sensitivity check index
Rec: recovery
REE: resting energy expenditure
RER: respiratory exchange rate
RQ: respiratory quotient
RR: relative risk
SBP: systolic blood pressure
SD: standard deviation
SMM: skeletal muscle mass
TBW: total body water
TG0: fasting triglycerides
VCO2: carbon dioxide production
VE: minute ventilation
VE/VCO2: ventilatory efficiency
VO2: oxygen consumption
VO2max: maximum oxygen consumption at peak exercise obtained through CPET
VO2peak: oxygen consumption at the point of maximum effort
WHtr: waist‐to‐height ratio

## 1 Introduction

Type 1 diabetes is an autoimmune disease characterized by the destruction of pancreatic beta cells and its capacity to secrete insulin. In 2021, there were 8.4 million individuals worldwide with type 1 diabetes. The prevalence is projected to increase to 13.5–17.4 million by 2040 [1]. These individuals are at risk of cardiovascular outcomes and premature mortality [2, 3]. This excess risk tends to be underestimated with the current cardiovascular scores [4]. Cardiorespiratory fitness (CRF) has been proposed by the American Heart Association as a new vital sign given its capacity to predict cardiovascular outcomes and all‐cause mortality [5, 6]. The maximal oxygen consumption (VO2max) is the surrogate measure of CRF; the gold standard test for its evaluation is the cardiopulmonary exercise test (CPET) with incremental protocols when maximum effort is achieved [7, 8]. The Bruce protocol is a standardized protocol that measures physiological responses to exercise and, when coupled with indirect calorimetry, provides a direct evaluation of VO2max [9, 10]. The VO2max is derived from the Fick equation and refers to the maximum capacity of the body to deliver oxygen to muscles during movement. The VO2max has non modifiable biological determinants such as sex, age and certain genetic variants [11, 12]. Body composition components also have an important contribution to VO2max, with a direct relation between muscle mass and an inverse relation between fat mass and VO2max [13, 14, 15]. The cell integrity and cell hydration measured through phase angle have a positive correlation with VO2max [16, 17, 18]. In addition, there are conditions that adversely alter VO2max such as diseases affecting ventilatory function (e.g., COPD, asthma) and cardiac function (heart failure) [19, 20, 21]. The diseases that affect glucose metabolism may influence VO2max, producing an altered response during an incremental CPET [22, 23, 24, 25]. Interestingly, cardiometabolic risk factors particularly those associated with insulin resistance, such as prediabetes, type 2 diabetes, hypoalphalipoproteinemia and obesity have also been independently associated with reduced VO2max [25, 26, 27]. However, VO2max is a trainable metric and can potentially be improved with an exercise program.

In addition to VO2max, CPET provides an array of metrics related to cardiovascular, ventilatory, and metabolic functions at four key stages: active rest, anaerobic threshold (AT), maximum effort (max), and recovery. Metrics such as the respiratory exchange ratio (RER) permit the evaluation of the use of energy substrates during different intensities of exercise. In the aerobic phase of exercise, ATP needed is obtained via oxidative phosphorylation and depends on the O2 delivered through the cardiopulmonary system and the use of lipids as energy fuel. In contrast, the anaerobic phase is extensively oxygen‐independent and relies on the capacity of muscle to obtain ATP via anaerobic glycolysis and may be affected if muscle glucose availability is altered [12, 28]. These physiological aspects highlight the importance of not only investigating oxygen consumption but also substrate utilization in individuals with diabetes. Previous evidence consistently demonstrates that individuals with type 1 diabetes show a reduction in VO2max [29, 30, 31]. The determinants and possible mechanisms of this reduction are not consistent among studies, with some studies demonstrating that metrics associated with diabetes control such as glycosylated hemoglobin (HbA1c) and glucose levels are mediators of the VO2max reduction while others are not [32]. We aimed to assess the influence of body composition, energy substrate use, as well as the inherent characteristics of the disease such as insulin doses and blood glucose control, during a cardiopulmonary exercise test in subjects with uncomplicated, long‐term type 1 diabetes.

## 2 Materials

### 2.1 Study Design and Recruitment

This was a cross‐sectional case–control study. Subjects were recruited between July 2023 and December 2024 through an open invitation on social media and referrals from the type 1 diabetes outpatient clinic of a tertiary level hospital in Mexico City. Subjects aged between 18 and 65 years were recruited. Exclusion criteria included established pulmonary or cardiovascular disease, pregnancy, severely uncontrolled diabetes (i.e., HbA1c ≥ 10%, 86 mmol/mol), history of back, hip, knee, or foot pathology including diabetic foot and symptomatic peripheral neuropathy that hindered walking or jogging on a treadmill, chronic kidney disease with an estimated glomerular filtration rate < 60 mL/min/1.73m^2^ and hospitalization within 3 months of the visit. The study was performed in accordance with the Declaration of Helsinki and all procedures were approved by the Institutional Review Board and Ethics Committee of the Instituto Nacional de Ciencias Médicas y Nutrición Salvador Zubirán (REF 4630). A sample size of 28 subjects (14 per group) was calculated with a formula for paired (dependent) means, to detect at least a 15% difference in VO2max between groups, with 90% power, and a significance level of 0.05, based on data from the study by McCarthy et al. describing reduced VO2max in persons with type 1 diabetes compared with a control group (peak VO2 32.55 vs. 42, 67 mL/kg/min) [33].

### 2.2 Visit Summary

Written informed consent was obtained at the beginning of each visit. A history of cardiovascular disease, arterial hypertension, dyslipidemia, current medications, tobacco and alcohol use was obtained. The doses of basal and rapid‐acting insulin, as well as insulin pump use were recorded. The insulin regimen and pharmacologic management were prescribed by the attending physician of the outpatient endocrinology clinic, based on the recommendations of the American Diabetes Association (ADA). Biochemical measurements were obtained within 2 months of the visit (fasting plasma glucose, HbA1c, liver function test, creatinine, and lipid profile). Physical activity was assessed through the International Physical Activity Questionnaire 9 (IPAQ‐9). All subjects underwent body composition analysis and indirect calorimetry at rest and during exercise. All subjects underwent a treadmill CPET.

### 2.3 Anthropometry and Body Composition Analysis

The height of subjects was obtained using a floor stadiometer (SECA). Waist and hip circumferences were obtained using anthropometric measuring tape. Body composition was assessed via bioelectrical impedance analysis (BIA) with a SECA mBCA514 medical body composition analyzer. BIA analysis included: weight (kg), fat mass (%, FM), fat‐free mass (%, FFM), skeletal muscle mass (%, SMM), and phase angle (°). The fat‐free mass to fat mass ratio (FFM/FM) was calculated by dividing the percentage of fat‐free mass by fat mass. The body mass index (BMI) was calculated as weight in kilograms divided by the square of the height in meters.

### 2.4 Indirect Calorimetry

The environmental conditions (temperature and humidity), gases, and volume of the gas analyzer are calibrated once a day. A breakfast of less than 400 kcal without caffeine followed by a fasting period of 6 h was mandatory prior to calorimetry. Subjects were fitted with a hermetic mask coupled to a pneumotachograph (Ganshorn Medizin Electronic). Heart rate was monitored via a 12‐lead electrocardiogram (ECG) with electrodes placed in the Mason and Likar distribution. Subjects were placed supine in a room with minimal visual and auditory stimuli. Following a rest phase of 20–30 min to achieve a basal state, VO2, VCO2, and heart rate were recorded at 10‐s intervals for 20 min using a gas analyzer (PowerCube, Ganshorn Medizin Electronic). Resting energy expenditure, REE (kcal/day) was calculated, discarding the values of VO2 and VCO2 from the first 5 min. Weir equation [34]

(1) $$\begin{array}{c} \mathrm{REE}=\left(1.106\times \mathrm{VCO}2+3.941\times \text{VO2}\right)\times 1.44\\ \mathrm{REE}=1.106\times \mathrm{VCO}2+3.941\times \mathrm{VO}2\times 1.44 \end{array}$$

The mean VCO2 and VO2 (mL/min) were used to calculate the respiratory quotient RQ as:

(2) RQ=VCO2/VO2RQ=VCO2/VO2

### 2.5 Cardiopulmonary Exercise Test

CPET was performed using a Cardiovit CS‐200 Ergo‐Spiro stress test system (Schiller) coupled to a motorized treadmill (TMX 428, Schiller) and gas analyzer (PowerCube, Ganshorn Medizin Electronic). The system was calibrated daily for atmospheric conditions using a thermohydrometer, volumes using a 2 L syringe, and gases with a gas mixture composed of 5% carbon dioxide (CO_2_), 16% oxygen (O_2_), and 79% nitrogen (N_2_). Baseline 12‐lead ECG and spirometry were obtained for all subjects to screen for arrhythmia or respiratory pathology before starting the test. The Bruce protocol was used [10] The protocol included a 40‐s active rest phase for breath stabilization. Participants without previous treadmill experience were given a one‐minute stabilization walking phase at 0.5 km/h and 0% incline. The traditional Bruce protocol consists of 7 incremental 3‐min stages: (1) 2.7 km/h, 10% incline, 4.7 METS; (2) 4.0 km/h, 12% incline, 6.8 METS; (3) 5.4 km/h, 14% incline, 9.1 METS; (4) 6.7 km/h, 16%, 12.9 METS; (5) 8.0 km/h, 18% incline, 15 METS; (6) 8.8 km/h, 20% incline, 16.9 METS; and (7) 9.6 km/h, 22% incline, 19.1 METS.

Throughout the CPET, subjects were monitored via continuous 12‐lead ECG and automatic blood pressure measurements at 3‐min intervals (BP‐200 plus, Schiller). The rate of perceived exertion was assessed with the modified Borg scale at 1‐min intervals. Active use of the treadmill handrails was not allowed unless the participant showed an unstable gait. CPET was terminated upon subject request due to exhaustion or at the discretion of the physician based on ECG changes indicative of arrhythmia or myocardial ischemia, a drop in systolic blood pressure ≥ 20 mmHg, loss of coordination, or any other indication where the subject's safety could be compromised. Once maximum exertion was achieved, the test ended with a 5‐min recovery stage (i.e., 2 km/h walk and 0% incline).

Cardiovascular, respiratory, and metabolic variables at active rest, anaerobic threshold, peak exercise, and recovery were automatically calculated by the LF8 software (version 8.5 M SR3; Ganshorn Medizin Electronic). The anaerobic threshold was calculated with the V‐slope method defined by a computerized linear regression analysis of the CO2 output flow plotted as a function of oxygen uptake, which is thought to detect the beginning of excess CO2 output generated from the buffering of H^+^ arising from lactic acid [35]. Maximum oxygen consumption (VO2max) was determined when oxygen consumption no longer increased despite increasing the workload (i.e., a plateau was reached). If a plateau was not reached, the VO2max was determined as the oxygen consumption at the point of maximum effort (also known as VO2peak). Wasserman plots were monitored throughout CPET. Only tests with maximal effort criterion, defined as a respiratory RER at peak exercise ≥ 1.0, were included in the analysis [36].

### 2.6 Statistical and Mathematical Analysis

Data normality was assessed with the Shapiro–Wilk skewness and kurtosis test for all variables. Variables with non‐normal distribution were expressed as median (interquartile range) while normal distribution variables were expressed as mean and standard deviation (SD). Categorical variables were expressed as percentages.

The determinants of VO2 and ventricular responses were found by multivariate regression (minimum square linear). In the linear approach, each variable y is related to the physiological variable x through:

(3) y=mx+y0y=mx+y0

where *m* is the slope and y0 is the *y* value at the origin.

Adjusted‐R was investigated for variables in a univariate and multivariate regression model to determine its independent influence on VO2max. All analyses were performed using STATA version 15.0 (StataCorp, College Station, Tx, USA) and figures using PRISMA 10.

In order to explore causality, we did a causality analysis with the method of Barron and Kenny (MEDSEM), considering oxygen consumption at maximum effort (VO2max, ml/kg/min) as the dependent variable and carbohydrate oxidation, phase angle and heart rate reserve (HRR) as mediators, and diabetes status (T1 diabetes/control subjects) as the main explanatory variable (binary independent variable) for males.

For female we did the causality analysis considering oxygen consumption at maximum effort (VO2max, ml/kg/min) as the dependent variable, and carbohydrate oxidation, heart rate reserve, visceral fat and glutamyl aminotransferase as mediators, and diabetes status (T1 diabetes/control subjects) as the binary independent variable.

## 3 Results

A total of 115 subjects were recruited from July 2022 to December 2024. Seven patients were eliminated for hypo‐hyperglycemia before the CPET (test differed) or for not meeting maximum effort criteria (Figure S1). One hundred eight subjects were included in the analysis (52% female), 54 with type 1 diabetes and 54 controls paired by age (± 2 years) and sex. The median age was 39 [28, 29, 30, 31, 32, 33, 34, 35, 36, 37, 38, 39, 40, 41, 42, 43, 44, 45, 46] years for type 1 diabetes and 39 [28, 29, 30, 31, 32, 33, 34, 35, 36, 37, 38, 39, 40, 41, 42, 43, 44, 45, 46, 47] for controls. 56% and 54% of participants had normal weight, 39% and 41% overweight and 5% and 5% had obesity, respectively (ns). The duration of disease was 21 ± 10 years. Insulin pump was used by 3 patients, and the rest were on a basal/prandial regimen. The prandial bolus was calculated according to the carbohydrate counting technique. The total daily dose of insulin was 0.61 ± 0.19 units/kg and 24% and 15% were in treatment with metformin and SGLT2‐I, respectively. The mean HbA1c was 7.9 (7.3%–8.7%), 63 (56–72 mmol/mol) in persons with diabetes and 5.2 (5.1%–5.5%), 33(32–37 mmol/mol) in persons without diabetes (*p* < 0.01). Total cholesterol levels were 154 (134–0.180) and 177 (153–206) mg/dL in persons with and without diabetes (*p* < 0.01). C‐LDL was 84 (66–106) mg/dL and 109 (91–137) mg/dL (*p* < 0.01). HDL cholesterol was 54 (47–67) and 48 (43–59) mg/dL (*p* < 0.01). Triglycerides were 75 (60–94) and 116 (84–0.150) mg/dL, respectively, (*p* < 0.01). AST was 18 (15–24) and 21 (17–25) UI/L, (*p* 0.06). ALT was 20 (13–23) and 21 (16–24) UI/L, (*p* 0.07). GGT was 16 (13–30) and 16 (12–26) UI/L, (*p* 0.040) and creatinine was 0.8 (0.7–0.9) and 0.9 (0.8–1.03) (*p* 0.07), respectively. 57% of the individuals with type 1 diabetes use statins or another lipid‐lowering agent. The baseline physical activity was 1793 (840–3066) vs. 2133 (1320–3408) METs/week, (*p* 0.40). These data are shown in Table 1.

**TABLE 1:** Baseline demographics and biochemical panel of the study population.

	Control *N* = 54	Type 1 diabetes *N* = 54	*p*
Female *N* (%)	28 (52)	28 (52)	
Age (years)	39 (28–47)	39 (28–46)	
Normal weight (%)	54	56	0.9
Overweight (%)	41	39	0.9
Obese (%)	5	5	0.9
SBP (mmHg)	111 (99–120)	108 (101–117)	0.8
DBP (mmHg)	70 (63–75)	70 (66–72)	0.8
Diabetes characteristics			
Disease duration (years)		21 ± 10	
Total insulin dose (U/kg)		0.61 ± 0.19	
ISGLt‐2 agents use (%)		15	
Metformin use (%)		24	
Statin use (%)	2	57	
METS/week ¶	2133 (1320–3408)	1793 (840–3066)	0.4
Fasting glucose (mg/dL)	88 ± 8	158 ± 63	< 0.01
HbA1c (%)	5.2 (5.1–5.5)	7.9 (7.3–8.7)	< 0.01
HbA1c (mmol/mol)	33 (32–37)	63 (56–72)	
Total cholesterol (mg/dL)	177 (153–206)	154 (134–180)	< 0.01
LDL‐C (mg/dL)	109 (91–137)	84 (66–106)	< 0.01
HDL‐C (mg/dL)	48 (43–59)	54 (47–67)	< 0.01
Triglycerides (mg/dL)	116 (84–150)	75 (60–94)	< 0.01
AST (UI/L)	21 (17–25)	18 (15–24)	0.06
ALT (UI/L)	21 (16–24)	20 (13–23)	0.07
GGT (UI/L)	16 (12–26)	16 (13–30)	0.40
Creatinine (mg/dL)	0.9 (0.8–1.03)	0.8 (0.7–0.96)	0.07

*Note:* Values are expressed as median (IQ range 25–75) or mean ± SD.

Abbreviations: ¶: assessed by IPAQ‐9, ALT: alanine amine transferase, AST: aspartate amine transferase, DBP: diastolic blood pressure, GGT: gamma glutamyl transferase, HDL‐C: high density lipoprotein, METS: metabolic equivalent = 3.5 mL O2/kg/min. LDL‐C: low density lipoprotein, SBP: systolic blood pressure.

Regarding anthropometry, BMI was 24.7 ± 3.2 vs. 24.9 ± 31, (*p* ns); Waist to hip ratio 0.85 ± 0.08 vs. 0.84 ± 0.07, (*p* ns), waist to height ratio 0.50 (0.45–0.55) vs. 0.51 (0.46–0.53), (*p* ns); fat mass for women 34.9% ± 7.1% vs. 33.5% ± 5.3%, (*p* ns); in men 21.2 ± 8.6 vs. 23.1 ± 6.8, (*p ns*); in women FFM 65.1% ± 7.1% vs. 66.5% ± 5.3%, (*p* ns), in men 78.8% ± 8.7% vs. 77% ± 6.9%, (*p* ns); in women visceral fat 1.6 (1.2–2.2) vs. 1.7 (1.5–2), (*p* ns); in men 2 (1.2–2.8) vs. 2.7 (1.7–3.1), (*p* ns); in women SMM 16.6 (15–17.8) vs. 18.3 (15.5–19.2) kg, (*p* ns); in men 27.2 (24.6–29.5) vs. 27.8 (25.5–30.16) kg, (*p* ns); in women, phase angle 4.86 ± 0.56 vs. 5.11 ± 0.8, (*p* ns), in men 5.6 ± 0.67 vs. 6.16 ± 0.5 *p* 0.01; in women total body water 48 ± 4.8 vs. 49 ± 3.5, (*p* ns), in men 57 ± 6.4 vs. 56 ± 4.8, (*p* ns). The data are shown in Table 2.

**TABLE 2:** Anthropometry and body composition of the study population.

	Control (54)	Type 1 diabetes (54)	*p*
Height (cm)	165 ± 8	164 ± 9	0.2
Weight (Kg)	68 ± 12.2	66.9 ± 11.3	0.3
BMI (kg/m^2^)	24.9 ± 3.1	24.7 ± 3.2	0.8
Waist‐to‐hip ratio	0.84 ± 0.075	0.85 ± 0.085	0.5
Waist‐to‐height ratio	0.51 (0.46–0.53)	0.50 (0.45–0.55)	0.4
FM (kg)			
Women	20.8 ± 5.2	21.8 ± 6.4	0.3
Men	18.2 ± 7.14	16.6 ± 8.6	0.2
FM (%)			
Women	33.5 ± 5.3	34.9 ± 7.1	0.4
Men	23.1 ± 6.8	21.2 ± 8.6	0.4
FMI	7.2 ± 2.4	7.2 ± 3.2	0.9
FFM (kg)			
Women	41.7 (38.6–42.9)	38.6 (37.3–41.3)	0.2
Men	59.2 (54.5–62.3)	58 (54.2–61.9)	0.3
FFM (%)			
Women	66.5 ± 5.3	65.1 ± 7.1	0.4
Men	77 ± 6.9	78.8 ± 8.7	0.4
FFMI	17.5 ± 2.5	17.4 ± 2.1	0.6
FFM/FM			
Women	2 (1.7–2.5)	1.8 (1.5–2.4)	0.3
Men	3.1 (2.6–4.4)	3.8 (2.7–4.8)	0.2
Visceral fat (L)			
Women	1.7 (1.5–2)	1.6 (1.2–2.2)	0.6
Men	2.7 (1.7–3.1)	2 (1.2–2.8)	0.7
SMM (kg)			
Women	18.3 (15.5–19.2)	16.6 (15.17.8)	0.1
Men	27.8 (25.5–30.16)	27.2 (24.6–29.5)	0.5
Phase angle (°)			
Women	5.11 ± 0.80	4.86 ± 0.56	0.22
Men	6.16 ± 0.50	5.6 ± 0.67	0.01
Total body water (%)			
Women	49 ± 3.48	48 ± 4.8	0.3
Men	56 ± 4.8	57 ± 6.4	0.5

*Note:* Values are expressed as mean ± or median (IQ range 25–75).

Abbreviations: BMI: body mass index, FM: fat mass, FFM: fat‐free mass, FFMI: fat‐free mass index, FMI: fat mass index, SMM: skeletal muscle mass.

**FIGURE 1:**
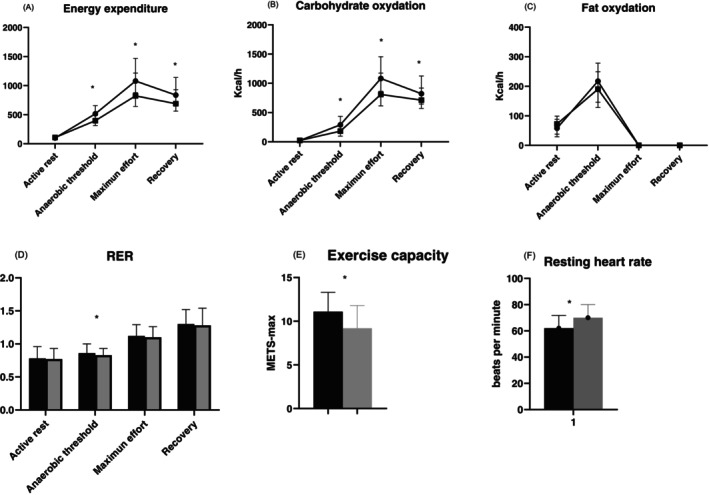
Indirect calorimetry at rest and during exercise. Black circle type 1 diabetes, black square control group. Black bar control and gray bar type 1 diabetes. *p < 0.01.

The resting energy expenditure (REE) adjusted by weight was 23.8 ± 0.55 and 23 ± 0.67 kcal/d/kg (*p* ns) in persons without and with type 1 diabetes. The respiratory quotient was 0.80 for type 1 diabetes and 0.79 for the control group (*p* ns). The carbohydrate oxidation (CHO‐ox) at rest was 22 (13–42) vs. 24 (9–42) (ns) during AT was 290 (178–435) vs. 183 (98–264) kcal/h (*p* < 0.01), and 1082 (863–1454) vs. 809 (614–1174) (*p* < 0.01) at maximum effort in persons without and with type 1 diabetes. The details of indirect calorimetry at rest and during exercise are shown in Figure 1 and Table S1.

**FIGURE 2:**
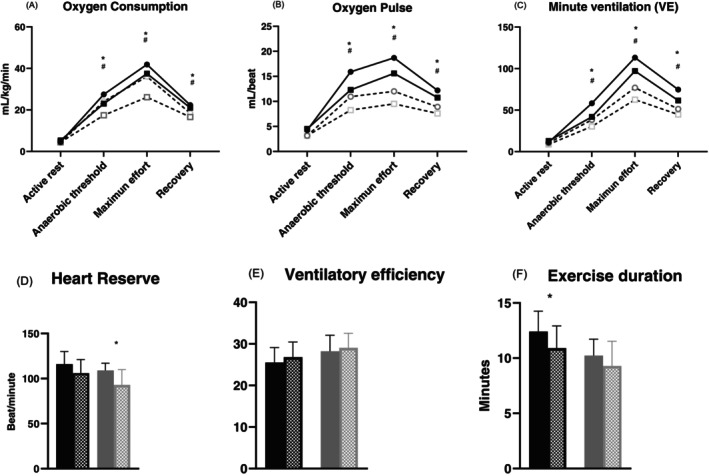
Cardiovascular, metabolic and ventilatory response during cardiopulmonary exercise test by sex. Black square men with type 1 diabetes, black circle men without type 1 diabetes. White square with dotted line women with type 1 diabetes, White circle with dotted line women without type 1 diabetes. Black column men without diabetes, black bar with dots men with type 1 diabetes and gray bar women without type 1 diabetes, gray bar with dots women with type 1 diabetes. *p < 0.01 for women, ^*#*^ *p* < 0.01 for men.

Heart rate reserve was 113 ± 16 and 101 ± 20 bpm (*p* < 0.01) in persons without and with type 1 diabetes, respectively. VO2 during the anaerobic threshold was 25.2 (22.3–30.8) vs. 19.1 (16.2–23.8) (*p* < 0.01), and maximum effort 39 ± 7.9 vs. 32 ± 9.2 (*p* < 0.01), respectively. The PuO2 was 12.7 (10.2–15.9) vs. 9.8 (8.1–12.3) (*p* < 0.01) during the anaerobic threshold, and 15.4 ± 4.7 vs. 12.5 ± 3.7 (*p* < 0.01) at maximum effort, respectively (Figure 2 and Table 3).

**TABLE 3:** Cardiovascular, metabolic and ventilatory responses during the cardiopulmonary exercise test.

	Control (54)	Type 1 diabetes (54)	*p*
Effort time (min)	11.3 ± 2	10.1 ± 2.3	< 0.01
HR‐Rest (bpm)	80 ± 16	90 ± 13	< 0.01
HR‐AT (bpm)	141 ± 22	131 ± 17	< 0.01
HR‐Max (bpm)	176 ± 16	171 ± 17	0.1
HR‐Max pred (%)	107 ± 9	105 ± 9	0.2
HR‐Rec (bpm)	134 ± 21	135 ± 17	0.8
HR Reserve (bpm)	113 ± 16	101 ± 20	< 0.01
METs Pred	10.2 ± 2.2	10.2 ± 2.2	0.4
METs Max	11.1 ± 2.2	9.2 ± 2.6	< 0.01
VO2‐Rest (mL/kg/min)	4.4 (3.4–5.8)	5 (3.9–6.1)	0.08
VO2‐AT (mL/kg/min)	25.2 (22.3–30.8)	19.1 (16.2–23.8)	< 0.01
VO2‐Max (mL/kg/min)	39 ± 7.9	32 ± 9.2	< 0.01
VO2‐Rec (mL/kg/min)	21 ± 3.5	19 ± 3.6	< 0.01
VO2 AT (% VO2Max)	69 ± 9	64 ± 9	< 0.01
PuO2‐Rest (mL/beat)	3.4 (2.9–5)	3.6 (3–4.5)	0.5
PuO2‐AT (mL/beat)	12.7 (10.2–15.9)	9.8 (8.1–12.3)	< 0.01
PuO2‐Max (mL/beat)	15.4 ± 4.7	12.5 ± 3.7	< 0.01
PuO2‐Rec (mL/beat)	9.8 (8.5–12.1)	8.7 (7.4–10.9)	< 0.01
RER AT	0.87 ± 0.06	0.83 ± 0.07	0.02
RER—Max	1.13 ± 0.06	1.10 ± 0.01	0.1
VE/VCO2	26.9 ± 3.9	27.9 ± 3.7	0.2
BR‐Rest (%)	91 (87–92)	90 (87–92)	0.08
BR‐Max (%)	16 (9–24)	28 (19–42)	< 0.01

*Note:* Values are expressed as mean ± SD or median (IQ range 25–75).

Abbreviations: AT: anaerobic threshold, BR: breathing reserve, CHO‐ox: carbohydrate oxidation, HR: heart rate, METS: metabolic equivalent = 3.5 mL O2/kg/min, Pred: predicted values by Wasserman formula, PuO2: oxygen pulse, Rec: recovery, RER: respiratory exchange rate, Rest: refers to active rest, VCO2: carbon dioxide production, VE/VCO2: ventilatory efficiency, VE: minute ventilation, VO2: oxygen consumption, with the subject standing on treadmill prior to the start of exercise.

**FIGURE 3:**
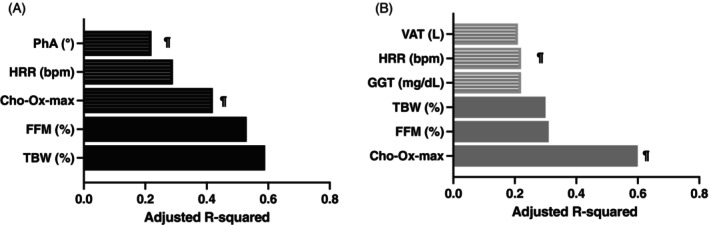
Impacting factors of VO2max in patients with type 1 diabetes by sex. (A) Men black bars with stripes are variables only significant in the diabetes group. (B) Women gray bars with stripes are variables only significant in diabetes group. ¶ Variables with partial or complete mediation effect.

The multivariate linear regression analysis by sex showed that VO2max had different determinants according to sex. In men, total body water (TBW) (adjusted R 0.59), FFM (adjusted R 0.53), carbohydrate oxidation at maximum effort (adjusted R 0.41), heart rate reserve (HRR) (adjusted R 0.29) and phase angle (adjusted R 0.22) were the main determinants, while in women carbohydrate oxidation at maximum effort (adjusted R 0.60), FFM (adjusted R 0.31), TBW (0.30), gamma‐glutamyl transpeptidase (GGT) (adjusted R 0.22), visceral adipose tissue (VAT) (adjusted R 0.21), and heart rate reserve (adjusted R 0.22) determined the variance in VO2max (Figure 3). Notably, other variables related to type 1 diabetes, including duration of disease, glycosylated hemoglobin, fasting glucose and insulin dose, did not show a significant association with CRF reduction. The influence of CHO‐ox on VO2max was confirmed with the Spearman correlation analysis that showed that carbohydrate oxidation has a strong correlation with VO2max in the type 1 diabetes group (*r* 0.79, *p* < 0.01).

**FIGURE 4:**
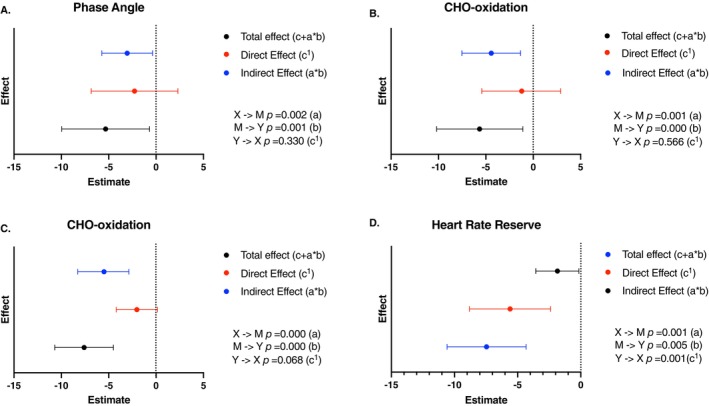
Estimate plots of mediation analysis in persons with type 1 diabetes. (A, B): men; (C, D): women.

The mediation analysis showed that phase angle and carbohydrate oxidation had a complete mediation effect. About 57% of the effect of diabetes on VO2max was mediated by phase angle, and 78% of the effect of diabetes on VO2max was mediated by carbohydrate oxidation. In women about 25% of the effect of diabetes on VO2max was mediated by heart rate reserve while 73% of the effect of diabetes on VO2max was mediated by carbohydrate oxidation (Figure 4).

## 4 Discussion

The purpose of this study was to assess the influence of body composition, energy substrates use, as well as the inherent characteristics of the disease such as insulin doses and blood glucose control, during a cardiopulmonary exercise test in subjects with uncomplicated, long‐term type 1 diabetes. They were paired by age and sex with persons without type 1 diabetes.

We found that VO2max was 8.4% lower in men with type 1 diabetes vs. paired control. In women the difference was greater with VO2max 27% lower in the group with diabetes. According to the linear regression models, VO2 at maximum effort was mainly governed by elements of body composition such as fat‐free mass (FFM) and the ratio between FFM and fat mass (FFM/FM) in men, while carbohydrate oxidation during the CPET was the main determinant in women independently of the disease status. The total body water percentage had an important impact in the four groups and was the main determinant of VO2max in men with diabetes. However, we found that persons with diabetes have specific determinants according to sex. In men with type 1 diabetes, the heart rate reserve explained 29% and phase angle explained up to 22% of the variance of VO2max. In women, heart rate reserve explained 22%, gamma glutamyl transpeptidase (GGT) explained 22% and visceral adipose tissue (VAT) up to 21% of VO2max (Figure 3). Interestingly, the mediation analysis showed that carbohydrate oxidation had a complete and strong mediation effect in both groups (78% and 73% for men and women, respectively). The role of phase angle was confirmed with a complete mediation effect in men while in women the heart rate reserve showed a partial mediation effect. This analysis discarded a possible indirect mediation effect for heart rate reserve in men and GGT and VAT in women.

Although it has been demonstrated that there are differences in body composition between persons with type 1 diabetes and healthy controls [37], our study showed no differences in muscle mass, fat mass, fat‐free mass, and its relation (FFM/FM). In line with our findings, previous evidence using dual energy X‐ray absorptiometry (DEXA) showed that muscle mass and fat mass were similar in adults with long‐standing type 1 diabetes in comparison with healthy controls [38].

The baseline level of physical activity was similar between groups and had no influence on the performance of the CPET. The reduction in VO2max in persons with type 1 diabetes has previously been described. Moser et al., reported that poor glycemic control was related to less economical use of oxygen at sub‐maximal work and an earlier time to exhaustion in a cohort of 64 subjects with type 1 diabetes [32]. Eickstein et al. showed that the difference in VO2max between individuals with type 1 diabetes and matched controls was 10 mL/kg/min. They also demonstrated that oxygen pulse and heart rate reserve were decreased, and that HbA1c was not associated with the outcomes evaluated [33]. In contrast, McCarty et al. with a single group design of 45 persons with long‐standing type 1 diabetes using insulin pump, reported that HbA1c was an important determinant of VO2 and that for every 1% increase in HbA1c there was a decrease of 3.5 mL/kg/min in the VO2max. They also showed that increases in HbA1c were associated with a reduction in oxygen pulse [39].

In our study VO2max was associated with carbohydrate oxidation in women with and without diabetes and explained up to 63% and 60% of the variance of VO2max, respectively (Figures 3 and 4). In men with diabetes 43% of VO2max variance was explained by carbohydrate oxidation while in men without diabetes, it did not have a significant influence. Turinese et al., demonstrated that RER and lactic acid levels at maximum effort during a CPET were lower in persons with type 1 diabetes in comparison with healthy controls, which was associated with a worse exercise tolerance, They argued that these differences were attributable to an altered use of energy substrates in favor of more lipid utilization in individuals with type 1 diabetes [30]. As exercise intensity increases, oxygen consumption also rises, requiring metabolic adaptations. In the aerobic phase, when the exercise intensity is low to moderate, substrate utilization is predominantly lipids. As intensity increases, carbohydrate utilization increases as well and exceeds the use of fatty acids in a process known as metabolic switch or metabolic flexibility, which generally coincides with the start of the anaerobic phase. This depends on sympathetic nervous system activity and the capacity of the musculoskeletal system to utilize the available glucose [40, 41]. The glucose availability relies on glycogen reserves and the capacity of muscle cells to uptake glucose by insulin‐dependent and independent mechanisms [42]. Any alteration in glucose utilization will cause a decrease in ATP production during the anaerobic phase and fatigue, which is the cause of premature termination of the effort. Our results suggest impairments in metabolic flexibility that in the setting of persons with long‐standing type 1 diabetes may be due to insulin resistance and poor glucose availability during intense exercise [28, 43, 44]. Remarkably, the prevalence of metformin use in our population was 24%. The development of insulin resistance in persons with type 1 diabetes is associated with the risk of vascular events and mortality [45].

Our study demonstrated that phase angle has important implications in VO2max and CPET performance in men with diabetes. Previously, phase angle, has been shown to be an independent predictor of VO2max and higher phase angle is positively associated with favorable CRF [16, 46].

The characteristics related to type 1 diabetes such as glucose control, duration of disease and insulin doses were not associated with VO2max or other outcomes evaluated.

Other findings that have the potential for future research were a significant decrease in the heart reserve of 11 beats per minute in persons with diabetes. An altered response in the heart rate has been reported before and may be attributable to defects in the sympathetic response to exercise [31, 47].

This study has some limitations. Firstly, body composition was not evaluated with dual energy x‐ray absorptiometry (DEXA), which is the gold standard [48]. However, bioelectrical impedance analysis correlates well with DEXA and is more readily available in the clinical setting [49]. Second, our study was made up of a cohort of persons between the 4th and 5th decades of life with long‐standing type 1 diabetes; for this reason our results cannot be extrapolated to younger and recently diagnosed type 1 diabetes. Thirdly, the glucose levels were monitored only at the beginning and at the end of the exercise, hindering a deeper understanding of glucose behavior during the exercise. The last meal before the CPET was not controlled and could have implications for CPET performance. Finally, lactic acid levels were not measured, which would have been useful to confirm the altered adaptation to the anaerobic phase in persons with type 1 diabetes.

The overarching strength of this study is the use of ergoespirometry, the gold standard for VO2max evaluation. Additionally, the tests were performed on a treadmill that permits the movement of most muscle groups, allowing for a more precise VO2max measurement. Most of the studies published in populations with type 1 diabetes are carried out with cycle ergometers potentially underestimating the VO2max. We used the Bruce protocol, which is standardized and widely used in research and clinical settings, promoting interpretability and comparability.

As conclusion, our results show that the mechanisms that prompt VO2max reduction in persons living with type 1 diabetes are mediated by components of body composition as fat‐free mass, body water proportion which has also been described in persons without diabetes. However, carbohydrate oxidation was a major determinant in middle‐aged persons with long‐standing type 1 diabetes regardless of sex. The association and the complete mediation effect of carbohydrate oxidation on VO2max was demonstrated by several approaches. We found specific factors for persons with type 1 diabetes as phase angle and in men and heart rate reserve in women. VO2max is a trainable metric, and our results highlight the importance of improving body composition and metabolic health as part of the integral management of long‐standing type 1 diabetes.

## Conflicts of Interest

The authors declare no conflicts of interest.

## Supporting information


**Table S1:** Indirect calorimetry: resting and during exercise.
**Table S2:** Effect sizes and confidence intervals of regression models.
**Figure S1:** Participant flow chart.

## Data Availability

The data that support the findings of this study are available on request from the corresponding author. The data are not publicly available due to privacy or ethical restrictions.
