# Supplementary material for: Impaired Cardiorespiratory Fitness in Type 1 Diabetes Is Associated With Metabolic Inflexibility and Specific Factors According to Sex
Source: J Diabetes. 2025 Dec 14;17(12):e70164. doi: 10.1111/1753-0407.70164 (PMC12703045; doi:10.1111/1753-0407.70164)
Supplement: Supplementary file 1 — Table S1: Indirect calorimetry: resting and during exercise. Table S2: Effect sizes and confidence intervals of regression models. Figure S1: Participant flow chart. [file JDB-17-e70164-s001.docx]

SUPPLEMENTARY MATERIAL

Table S1. Indirect Calorimetry: Resting and during exercise

|  | Control  N= 54 | Type 1 diabetes  N= 54 | *p*-value |
| --- | --- | --- | --- |
| REE kcal/day/kg | 23.87 ± 0.55 | 23 ± 0.67 | 0.3 |
| Basal RQ | 0.79 ± .05 | 0.80 ± .06 | 0.6 |
| Resting HR (bpm)* | 63 ± 10 | 70 ± 11 | <0.01 |
| Active rest HR (bpm)* | 80 ± 16 | 90 ± 13 | <0.01 |
| AT-EE kcal/h | 515 (415-656) | 398 (312-488) | <0.01 |
| Max-EE kcal/h | 1097 (878-1469) | 824 (642-1215) | <0.01 |
| Active rest – CHO-ox kcal/h | 22 (13-42) | 24 (9-42) | 0.6 |
| AT – CHO-ox kcal/h | 290 (178-435) | 183 (98-264) | <0.01 |
| Max effort– CHO-ox kcal/h | 1082 (863-1454) | 809 (614-1174) | <0.01 |
| Active rest – Fat-ox kcal/h | 58 (38-88) | 73 (52-99) | 0.2 |
| AT – Fat-ox kcal/h | 217 (146-278) | 190 (128-249) | 0.09 |
| Time for anaerobic glycolysis [Metabolic Switch] (minutes) | 6.10 ± 1.9 | 5.2 ± 1.6 | 0.01 |
| Time AT (minutes) | 6.9 ± 2.1 | 5.1 ± 1.7 | <0.01 |
| Pre-exercise glucose (mg/dL) | 89 ± 8 | 144 ± 48 | <0.01 |
| Post-exercise glucose (mg/dL) | 110 ± 19 | 152 ± 40 | <0.01 |
| Delta glucose (%) | 23 ± 23 | 10 ± 24 | <0.01 |

Table S1. Indirect calorimetry at rest and during exercise

Values are expressed as median (IQ range 25-75) or mean ± SD. REE: resting energy expenditure, RQ: respiratory quotient, HR: heart rate (mean HR measured over 20 minutes), EE: energy expenditure, Carbohydrates: CHO, Anaerobic threshold: AT, Respiratory Exchange Ratio: RER. *Resting HR: patient supine position 20 minutes, Active rest: Subject standing on treadmill before starting the test.

Table S2. Effect sizes and Confidence Intervals of Regression Models

| Variable | Adjusted R-squared | Coef | t-value | Confidence Interval | *p*-value |
| --- | --- | --- | --- | --- | --- |
| Men | | | | | |
| Phase Angle ¶ | 0.22 | 6.96 | 2.69 | 1.58-12.35 | 0.014 |
| Heart Rate Reserve ¶ | 0.29 | 0.25 | 3.25 | 0.09-0.40 | 0.004 |
| CHO-oxidation * | 0.42 | 0.016 | 4.37 | 0.008-0.024 | <0.001 |
| Free fat mass (%) * | 0.53 | 0.79 | 5.12 | 0.47-1.11 | <0.001 |
| Total body water (%) * | 0.59 | 1.12 | 5.72 | 0.71-1.53 | <0.001 |
| Women | | | | | |
| Visceral adipose tissue (L) ¶ | 0.21 | -4.78 | -2.84 | -8.24 - -1.31 | 0.009 |
| Heart Rate Reserve ¶ | 0.22 | 0.17 | 2.94 | 0.05-0.29 | 0.007 |
| Gamma-glutamyl transferase ¶ | 0.22 | -0.12 | -2.66 | -0.21 - -0.026 | 0.015 |
| Total body water (%) * | 0.30 | 0.74 | 3.50 | 0.30-1.17 | 0.002 |
| Free fat mass (%) * | 0.31 | 0.50 | 3.56 | 0.21-0.80 | 0.002 |
| CHO-oxidation * | 0.60 | 0.026 | 6.46 | 0.017-0.034 | <0.001 |

Table S2. Effect sizes and Confidence Intervals of Regression Models

¶: Determinants exclusive to persons with type 1 diabetes. *Statistically significant

Figure S1. Participant Flow Chart

Figure S1. Participant Flow Chart
